# Mortality Risk After Cardiac Surgery: Application of Inscor in a University Hospital in Brazil's Northeast

**DOI:** 10.5935/1678-9741.20160080

**Published:** 2016

**Authors:** João Vyctor Silva Fortes, Mayara Gabrielle Barbosa e Silva, Thiago Eduardo Pereira Baldez, Marina de Albuquerque Gonçalves Costa, Luan Nascimento da Silva, Renata Silva Pinheiro, Zullma Sampaio Fecks, Daniel Lago Borges

**Affiliations:** 1 Hospital Universitário da Universidade Federal do Maranhão (HUUFMA), São Luís, MA, Brazil; 2 Universidade Federal do Piauí (UFPI), Teresina, PI, Brazil.

**Keywords:** Cardiac Surgical Procedures, Mortality, Risk Factors

## Abstract

**Objective:**

To apply the InsCor in patients undergoing cardiac surgery in a university hospital in Brazil's northeast.

**Methods:**

It is a retrospective, quantitative and analytical study, carried out at the University Hospital of the Federal University of Maranhão. InsCor is a remodeling of two risk score models. It evaluates the prediction of mortality through variables such as gender, age, type of surgery or reoperation, exams, and preoperative events. Data from January to December 2015 were collected, using a Physical Therapy Evaluation Form and medical records. Quantitative variables were expressed as mean and standard deviation and qualitative variables as absolute and relative frequencies. Fisher's exact and Kruskal-Wallis tests were applied, considering significant differences when *P* value was < 0.05. Calibration was performed by Hosmer-Lemeshow test.

**Results:**

One hundred and forty-eight patients were included. Thirty-six percent were female, with mean age of 54.7±15.8 years and mean body mass index (BMI) equal to 25.6 kg/m^2^. The most frequent surgery was coronary artery bypass grafting (51.3%). According to InsCor, 73.6% of the patients had low risk, 20.3% medium risk, and only 6.1% high risk. In this sample, 11 (7.4%) patients died. The percentage of death in patients classified as low, medium and high risk was 6.3, 7.1% and 11.1%, respectively.

**Conclusion:**

InsCor presented easy applicability due to the reduced number of variables analyzed and it showed satisfactory prediction of mortality in this sample of cardiac surgery patients.

**Table:** 

Abbreviations, acronyms & symbols	
**BMI**	**= Body mass index**
**CABG**	**= Coronary artery bypass grafting**
**CKD**	**=Chronic kidney disease**
**CPB**	**=Cardiopulmonary bypass**
**ICU**	**=Intensive Care Unit**
**LL**	**=Lower limit**
**MV**	**=Mechanical ventilation**
**UL**	**=Upper limit**

## INTRODUCTION

Cardiovascular diseases contribute to high rates of morbidity and mortality in health systems. In Brazil, one third of deaths are a consequence of such diseases, leading to a significant increase in health costs^[1]^.

Cardiac surgery is widely used to treat those diseases ^[2]^. It is indicated to provide better quality of life to the patients. Among the surgeries performed, the most recurrent is coronary artery bypass grafting (CABG). About 90% of CABG patients present significant improvement in cardiac function and, consequently, better quality of life. However, despite advances in cardiac surgery in Brazil, success depends on adequate pre- and postoperative assistance^[3]^.

Application of risk scores as predictor of postoperative complication and mortality is well established, especially in cardiac surgery. Nevertheless, development and validation of local models are becoming increasingly necessary due to demographic, socioeconomic, and cultural aspects of different populations^[4,5]^. The combination of the data resulting from the risk score models allows for adaptation, improvement and even innovation in treatment programs^[2]^.

Nowadays, there are about 20 risk score models in cardiac surgery around the world. The most applied are: Parsonnet score, Society of Thoracic Surgeons risk score (STS score), Higgins score, Northern New England score (NNE score), Ambler score, 2000 Bernstein-Parsonnet score, and EuroSCORE. The last two can be used bedside for both CABG and valvar surgery^[6]^.

The InsCor risk score was developed at Instituto do Coração do Hospital das Clínicas da Faculdade de Medicina da Universidade de São Paulo (InCor-HCFMUSP) to predict mortality of patients undergoing CABG and/or valvar surgery at that hospital. It includes the following variables: age, gender, type of surgery, prior myocardial infarction, reoperation, ejection fraction, and preoperative events^[7]^. InsCor is a remodeling product of EuroSCORE and 2000 Bernstein-Parsonnet, that shows better applicability and greater ease of assistance care^[2,7]^.

In our institution, there is no mortality prediction risk model for patients undergoing cardiac surgery. Therefore, it is necessary to apply scores validated in other samples. Thus, in this study, the application of InsCor as predictor of mortality in patients undergoing cardiac surgery at a university hospital in Brazil's northeast was investigated.

## METHODS

This is a retrospective, quantitative and analytical study performed in a university hospital of São Luis, Maranhão, Brazil. Data were collected from the Physical Therapy Evaluation Form and medical records of patients who underwent cardiac surgery and were admitted to the Cardiologic Intensive Care Unit (ICU) between January and December 2015. Patients whose forms did not contain enough information for the study were excluded.

A form, developed exclusively for this study, was used to record the data. It included the following:

- Demographic and anthropometric data: gender, age, weight, height, and BODY MASS INDEX (BMI);
- Clinical data: clinical diagnosis and comorbidities;
- Surgical data: type of surgery, duration of surgery, cardiopulmonary bypass (CPB), and aortic clamp;
- Postoperative data: duration of mechanical ventilation, postoperative complications, length of stay in ICU, and death.

### InsCor

The score ranges from 0 to 30 and defines three categories of risk of mortality after cardiac surgery: low risk (0-3 points); medium risk (4-7 points) and high risk (> 8 points), as described by Mejia et al.^[7]^ (Table 1).

**Table 1:** InsCor.

Variables	Score
Age (> 70 years)	3
Gender (female)	2
CABG + valve surgery	2
Recent infarction (< 90 days)	2
Reoperation	3
Surgical treatment of aortic valve	2
Surgical treatment of tricuspid valve	3
Creatinine (> 2 mg /dl)	5
Ejection fraction < 30%	3
Events*	5

* Includes at least one of the following situations prior to surgery: intra-aortic balloon, cardiogenic shock, ventricular tachycardia or fibrillation, orotracheal intubation, acute renal failure, inotropic drugs, and cardiac massage. CABG=coronary artery bypass grafting

### Statistical Analysis

Data were evaluated using the Stata/SE statistical software version 12.0 (StataCorp, College Station, TX, USA) and Microsoft Office Excel 2013. Calibration was performed by correlation between observed and expected mortality through the Hosmer-Lemeshow test. Quantitative variables were described as means and standard deviation and their differences were verified using the Kruskal-Wallis test. Qualitative variables were expressed as absolute frequency and proportions. Association between categorical variables and outcomes were verified using Fisher's exact tests. Results were considered statistically significant when *P* value was < 0.05.

### Ethics and Informed Consent Form

This study was approved by the Institutional Ethics Committee under number 1.469.394, being exempted from the need for informed consent as explained by the type of design applied.

## RESULTS

One hundred and forty-eight patients were included. Of those, 11 (7.4%) died. Mean age was 54.7±15.8 years and 54 (36.5%) patients were female. Regarding the procedures, 76 (51.3%) patients underwent coronary artery bypass grafting, 65 (43.9%) underwent valve surgery and seven (4.8%), coronary bypass + valve surgery. There were six (4%) reoperations and three (2%) patients presented creatinine level > 2 mg/dl. Demographic, clinical, and surgical data are shown in Table 2.

**Table 2:** Demographic and clinical data of patients undergoing CABG.

Variables	n (%)	Mean (SD)
**Gender** Male Female	54 (36.5) 94 (63.5)	
**Age (years)**		54.7 (15.8)
**BMI (kg/m^2^)**		25.6 (4.2)
**Comorbidities (n)** Hypertension Diabetes mellitus Smoking Dyslipidemia Myocardial infarction Stroke CKD	95 (64.2) 47 (31.8) 19 (12.8) 14 (9.5) 8 (5.4) 6 (4.1) 5 (3.4)	
**InsCor**		
Low risk	111 (73.6)	
Medium risk	28 (20.3)	
High risk	9 (6.1)	

BMI=body mass index; CKD=chronic kidney disease; InsCor=mortality risk score

Mean risk score was 2.5±2.4. Low risk was found in 73.6% of patients, while 20.3% had medium risk and 6.1%, high risk. Percentage of death in each group was, respectively, 6.3%, 7.1% and 11.1%. Calibration of InsCor was adequate (*P*=0.116) (Table 3).

**Table 3:** Mortality risk score calibration by groups.

InsCor	Cases	% observed	95% CI		% predicted	95% CI	
			LL	UL		LL	UL
Low	111	6.3	1.8	10.8	6.8	2.1	11.5
Medium	28	7.1	- 2.4	16.6	6.8	-2.5	16.2
High	9	11.1	- 9.4	31.6	6.6	-9.6	22.8

95% CI= 95% confidence interval; LL=lower limit; UL=upper limit Hosmer-Lemeshow test (*P*=0.116)

Comparing surgical variables of risk categories, significant difference was found between low and medium risk, concerning CPB, aortic clamp, and surgery duration. Surgical data, mechanical ventilation duration, and ICU length of stay are shown in Table 4.

**Table 4:** Surgical data, mechanical ventilation duration, and length of stay at ICU of patients undergoing cardiac surgery, according to InsCor categories of risk.

Variables	Low	Medium	High	*P*
CPB duration (min)	90 (70; 110)	111.5 (88.7; 146.2)	115 (85; 230)	0.004*
Aortic clamp duration (min)	66 (44,5; 89)	88 (69.5; 113)	88 (65; 150)	0.003*
Surgery duration (min)	3.7 (3.1; 4.1)	4.3 (3.7; 4.7)	4.3 (3.2; 6.2)	0.002*
MV duration (hours)	14.3 (11.7; 18.3)	15.1 (8.2; 17.6)	14.5 (12.1; 24)	0.82
Length of ICU stay (days)	4 (3; 5)	5 (3; 7)	7 (3.7; 8.5)	0.02^#^

CPB=cardiopulmonary bypass; ICU=intensive care unit; MV=mechanical ventilation Data shown as median (q25; q75). Kruskal-Wallis test (Dunn’s post hoc)

* *P*<0.05 comparing low and medium risk;

# *P*<0.05 comparing low and high risk

## DISCUSSION

Patients undergoing cardiac surgery may present unsatisfactory post-operative evolution, with severe clinical conditions, requiring long hospital stay and, occasionally, with sequelea that can compromise their quality of life^[8]^.

Several models of risk scores have been developed in order to predict mortality in cardiac surgery^[9]^. These systems of risk prediction, based on preoperative data, allow for the stratification of patients and prior planning of intra and postoperative periods^[10]^. Some risk scores developed abroad are used in Brazil, such as EuroSCORE, which is widely accepted worldwide^[11]^.

InsCor is a national risk score based on the remodeling of two international risk scores: 2000 Bernstein-Parsonnet and EuroSCORE. Like previous studies, we found adequate calibration of InsCor. Mejía et al.^[11]^ analyzed the performance of the two aforementioned scores in a group of 744 patients at InCor-HCFMUSP and showed that both had proper calibration, with no difference between the expected and observed mortality. Garofallo et al.^[6]^ found similar results in a study performed at the Instituto de Cardiologia do Rio Grande do Sul (IC/FUC). Thus, it appears that the InsCor has adequate applicability in predicting mortality after cardiac surgery.

Lisboa et al.^[5]^ analyzed the performance of EuroSCORE II in 1000 patients undergoing cardiac surgery, comparing it to the InsCor and EuroSCORE. The authors concluded that only the last two were adequate in all stages of validation, due to mistakes in the design of EuroSCORE II, hence reinforcing the importance of a national risk score model.

It is expected that patients with higher mortality risk require a longer period of CPB and aortic clamping during cardiac surgery, as found in this study. Al-Sarraf et al.^[12]^ demonstrated a connection between age, ejection fraction < 50%, emergency surgery, and associated surgical procedures (CABG + valve) with prolonged CPB and aortic clamp duration. Such variables increase the risk of mortality according to InsCor.

Olmos et al.^[13]^ reported that factors such as BMI, smoking, and elderly age contribute to the occurrence of respiratory complications and consequent increase in hospital length of stay. Female gender, reoperation, heart valve surgery, and associated surgeries are also related to prolonged length of stay^[14,15]^. It is noteworthy that several of these variables are included in the InsCor assessment.

Although InsCor was proposed to verify the prediction of mortality, perhaps this score might be related to the occurrence of postoperative complications and prolonged length of stay. Therefore, other studies are suggested to clarify such points.

### Limitations

The short period of data collection, possibly an explanation for the small number of patients, particularly high-risk patients, is pointed as the main limitation of this study. Scarce publications about InsCor also made it difficult to discuss the results of this study.

## CONCLUSION

InsCor presented easy applicability due to the reduced amount of analyzed variables and showed satisfactory prediction of mortality in this sample of cardiac surgery patients.

**Table:** 

Authors’ roles & responsibilities	
JVSF	Conception and design study; manuscript redaction or critical review of its content; realization of operations and/ or trials; final manuscript approval
MGBS	Manuscript redaction or critical review of its content; realization of operations and/or trials; final manuscript approval
TEPB	Analysis and/or data interpretation; realization of operations and/or trials; final manuscript approval
MAGC	Analysis and/or data interpretation; manuscript redaction or critical review of its content; statistical analysis; final manuscript approval
LNS	Manuscript redaction or critical review of its content; realization of operations and/or trials; final manuscript
RSP	approval
	Analysis and/or data interpretation; statistical analysis;
ZSF	final manuscript approval
	Manuscript redaction or critical review of its content; final
DLB	manuscript approval
	Manuscript redaction or critical review of its content; final manuscript approval
